# Determination of Hansen solubility parameters of water-soluble proteins using UV–vis spectrophotometry

**DOI:** 10.1016/j.heliyon.2023.e21403

**Published:** 2023-10-29

**Authors:** Neveen AlQasas, Daniel Johnson

**Affiliations:** New York University Abu Dhabi, Division of Engineering, Abu Dhabi, United Arab Emirates

## Abstract

Determination of solubility parameters by dissolution tests are difficult for some valuable molecules, such as proteins, where the quantities available are small, UV–vis spectroscopy can determine dissolved concentrations of even small amounts of material, but accurate determination of dissolution is difficult in relatively poor solvents, due to difficulty with constructing a reliable calibration curve. In this work we report a new simple procedure to determine the relative dissolution of proteins in various solvents using UV vis spectroscopy for the determination of Hansen solubility parameters (HSP) of proteins. This method allows qualitative determination of the amounts of BSA dissolved in various solvents. The amounts of BSA dissolved in each solvent, can then be used to rank solvents as good or bad for HSP calculation purpose, which gives more reliable ranking than observation alone in dissolution tests. To be able to evaluate the HSP of any solid material, the solubility of the tested material in a range of solvents needs to be determined. Solvents are then scored as good or bad based on observations and from the known properties of those solvents, the HSP of the solute molecule in question can be calculated. Here, bovine serum albumin (BSA) was used as a model protein, but the procedure reported here can be applied to any soluble protein or other macromolecule with a clear UV/vis adsorption peak. This procedure requires the tested material to be highly soluble in water, therefore eliminating the need for the preparation of many standard solutions of BSA in different solvents. Only a set of standard solutions of protein in water is required. UV–vis spectroscopy was used to analyze the remaining solid resuspended in water after centrifugation of solutions of BSA dissolved in other solvents to separate any undissolved protein. The HSP of BSA obtained via this procedure was compared to values previously obtained for BSA using other methods. A very good agreement between the HSP obtained in this work with the that reported by Houen et al. which used amino acid analysis for the estimation of the solubility of BSA in various organic solvents.

## Graphical abstract

**Figure:**
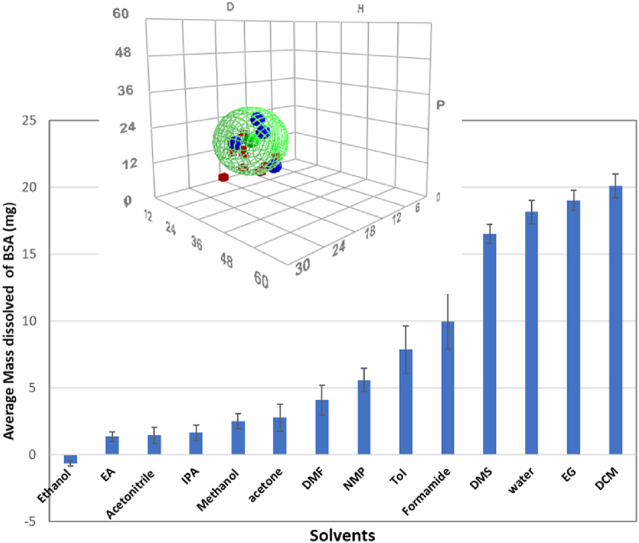

## 1 Introduction

The adsorption of proteins to surfaces is a very complex phenomenon dependent on various factors. Initial protein adsorption is predominantly determined by the protein concentration in the solution and the diffusion rates of the individual proteins. The interaction of proteins with solid surfaces involves a complex interplay of concurrent processes of adsorption, displacement and stabilization of protein - surface interactions due to conformational and structural changes [1]. Various theories have been developed to explain protein adsorption to surfaces, such as Berg's law and Whiteside's rules [2]. Hansen solubility parameters (HSPs) are another important set of parameters that can give an indication about the affinity of proteins to certain surfaces. It has been reported that the HSP distance between a polymer surface and bovine serum albumin (BSA) is inversely related to the natural logarithm of the amount of BSA adsorbed [3]. The Hansen solubility parameter (HSP) distance is a measure of a substance's affinity to known solvents based on the HSP of both substances. Knowing the HSP of a certain substance can give an indication about its compatibility or ability to be dissolved in another substance. The applications of HSP span a wide area, including optimizing solvent selection [[4], [5], [6]], improving polymer compatibility, enhancing pigment dispersion, understanding and controlling processes and, in general, offering guidance where the affinities between materials are of prime importance [[7], [8], [9], [10], [11]]. HSP also aids in substitution of solvents to create less hazardous formulations in various products, such as cleaning agents, printing inks and adhesives [[12], [13], [14]]. The Hansen solubility parameter had been also used widely for semiconductors and solar cell applications [[15], [16], [17]].

For determination of the HSP, methods using polymer statistics cannot be applied to most proteins because they adopt specific (native) conformations under different conditions [18]. There are few reports in the literature for the HSP of BSA, for instance. BSA solubilities were measured in different solvents by Houen et al. [19], which, although not calculated by Houen, can be used to estimate the HSP of the BSA protein. The BSA protein concentrations in Houen's work was obtained using amino acid analysis (AAA). The AAA method affords an absolute quantitative measure of protein content of a sample independent of an external protein reference standard [20]. Intrinsic viscosity measurement is another technique to obtain the HSP of any substance. Intrinsic viscosity measurements are very useful in assessing the interaction between the solute and solvent [18]. Curvale et al. [21] determined the intrinsic viscosity of BSA in aqueous solutions and found that the intrinsic viscosity is an expression of the interaction between biopolymer and solvent which reflects the solvent's ability to swell the macromolecule and found that BSA has a very low intrinsic viscosity value at pH 7.4. Masuelli [18] proposed the use of the intrinsic viscosity to concentration ratio given the difficulty of measuring the intrinsic viscosity at high protein concentration. UV–Vis and FTIR spectroscopy have also been used to measure the BSA concentration in aqueous solutions [22] by preparing BSA-water standard solutions and calibration curves. In the work of Carvalho et al. [23], HSP of different molecules was obtained via different three methods, the differential scanning calorimetry, intrinsic viscosity measurements, and ultraviolet visible spectroscopy (UV–Vis). The determination of HSP of a molecule using either intrinsic viscosity or the UV–Vis is based on the principle that the compound will have the same HSP of the solvent system in which the compound exhibits the highest interaction. In the case of the UV–vis, the absorbance of each system in the various solvents is measured, and the substance's HSP will have the same HSP as the solvent system in which the absorbance is the greatest, however this method will yield the total HSP parameter only, and not the specific dispersive, polar and hydrogen bonding of the material under investigation.

Inverse Gas Chromatography, has been used widely in measuring the Hansen solubilities of different solid materials [[24], [25], [26]]. However Inverse Gas Chromatography is an expensive instrument that may not be easily available. Most previous work measured either the solubility of BSA in water, its intrinsic viscosity and solubility in different solvents without calculating specifically the Hansen solubility parameter. Alternatively, methods have been developed to determine the solubility parameters of thin films from surface measurements using the contact angles of various solvents and calculating the HSP via surface free energy determination [27].

In this work, to allow the ranking of the various solvents and categorize them in terms of their ability to dissolve the protein under investigation, UV–vis was employed to determine quantities of protein dissolved in each solvent. Those rankings can then be used for calculation of HSP using the Hansen sphere procedure. Whereas direct determination using UV–vis would only allow the total solubility parameter to be determined, this method will allow determination of the individual polar, hydrophobic and dispersive component s of the HSP. Therefore, we propose a simple and inexpensive procedure that uses only UV–vis measurements to quantify the amounts of BSA dissolved in different organic solvents without the need for preparing many standard BSA-solvent samples. Here, BSA was used as a model protein, but this procedure could be generalized to any other proteins with high water solubility. HSP parameters can conventionally be calculated from a process of ranking the solubility in a number of solvents of known and diverse solubility parameters as either good or bad solvents [12]. Quantifying the dissolved BSA amounts aids in the ranking of good and bad solvents in a more reliable way than by simple observation of dissolution, which is problematic when only small amounts of a particular protein are available. Knowing the HSP of proteins and other biomacromolecules will help in determining the affinity for other materials and therefore the likely adsorption rates for surfaces manufactured from those materials. Fang et al. [3] reported a relation between the amount of protein adsorbed to polymer surfaces and the HSP distance between the protein and the polymer. This knowledge has potential to help in the selection of materials for various applications where adsorption of proteins to surfaces is important, such as the biocompatibility of medical devices and the fouling resistance of polymer membranes and marine coatings.

In order to accurately measure the BSA concentration in the different organic solvents using UV–Vis, standard samples of BSA in each of the solvents used (in our case 14 solvents) would conventionally need to be prepared, and therefore 14 different calibration curves are needed to be able to estimate the concentration of BSA in unknown samples. This is not only impractical, but also not feasible. Preparing standard samples with known concentration of BSA for each organic solvent requires BSA to be soluble in all the solvents. If a solvent cannot completely dissolve the BSA under experimental conditions, standard known-concentration samples cannot be prepared. This would preclude the obtaining of meaningful standard curves for any of the poor solvents required for the HSP calculation. To overcome this difficulty, and to be able to accurately assess the amount of BSA dissolved in each organic solvent, a new procedure based on the standard calibration curves obtained from standard samples of BSA in water only, is proposed. This method eliminates the need of making many standard samples and also uses an easy way to quantify the solubility using UV–Vis measurements only. This method can also be applied to any solid that is known to have a high solubility in water.

Hansen [28] indicated that the total cohesive energy of a compound is the sum of all energy contributions, which are due to a combination of dispersive, polar and hydrogen bonding interactions. HSP is based on the cohesive energy density related to three different parameters: dispersion forces (δd), permanent molecular bipolar forces (δp), and hydrogen bonding (δh). The sum of the squares of these three parameters give the square of the total (Hildebrand) solubility parameter (δt) or total cohesion energy (Eq (1.1)):

Eq. 1.1 $$\delta_{t}^{2}=\delta_{D}^{2}+\delta_{P}^{2}+\delta_{H}^{2}$$

The degree of affinity between two different materials can be found by comparing their HSP values. The correlation between the HSP values of two materials is a measure known as the HSP distance, typically designated as R_a_. The smaller the R_a_ the more compatible those materials are. Hansen and Skaarup [29] developed an equation to calculate the solubility parameter distance between two materials using the following relation:

Eq. 1.2 $$R_{a}^{2}=4{\left(\delta_{d1}-\delta_{d2}\right)}^{2}+{\left(\delta_{p1}-\delta_{p2}\right)}^{2}+{\left(\delta_{h1}-\delta_{h2}\right)}^{2}$$

Eq. (1.2) was developed empirically and has been found to be able to accurately represent the solubility data where $\delta_{d},\delta_{p},\delta_{h}$ are the center of a sphere plotted in cartesian space.

The distance R_a_ in Eq. (1.2) should not exceed a certain radius of interaction R_o_ in the solubility sphere of a polymer for good solvents. A ratio defined as the relative energy density (RED) as shown in equation (1.3) is a good tool for quick evaluation of whether a solvent is likely to appear inside the solubility sphere of a polymer [12,28].

Eq. 1.3 $$RED=\frac{R_{a}}{R_{o}}$$

## 2 Materials and methods

### 2.1 Materials and chemicals

BSA was purchased from Sigma Aldrich. Fourteen solvents were used to determine the HSP values of BSA protein using solubility measurements: N-Methyl-2-pyrrolidone (NMP), dimethyl formamide (DMF), dimethyl sulfoxide (DMS), ethylene glycol, ethyl acetate, acetone, ethanol, methanol, isopropyl alcohol (IPA), water, acetonitrile, dichloromethane (DCM), toluene, and formamide. All chemicals were obtained from Sigma Aldrich. All water used in these experiments was high purity water (18.2 MΩ cm) using a Milli-Q high purity water system.

### 2.2 Instrumentations

A Thermo Scientific Multifuge X3R (42435919) was used to centrifuge all samples at 4000 rpm at 20^ο^C for 15 min. A UV-3100 PC visible light spectrophotometer was also used to analyzed the remaining BSA solid after centrifugation.

## 3 Methodology

The amount of BSA dissolved in each organic solvent was quantified using UV–Vis Spectrophotometry. A measured amount of BSA protein was dissolved in 14 different solvents. Each vial was kept under continuous stirring for 24 h and then centrifuged for 15 min under ambient temperature and supernatant was removed. Remaining solids were vacuum dried to ensure the complete evaporation of solvents. The remaining solids were re-dissolved in a known volume of water and analyzed using UV–Vis and the resultant concentration was quantified based on the BSA-water calibration curves. Based on the UV–vis analysis, solvents were ranked accordingly and the HSP of BSA was obtained using the software developed by the Hansen group, HSPiP [30].

### 3.1 Preparation of calibration curves

**Fig. 1:**
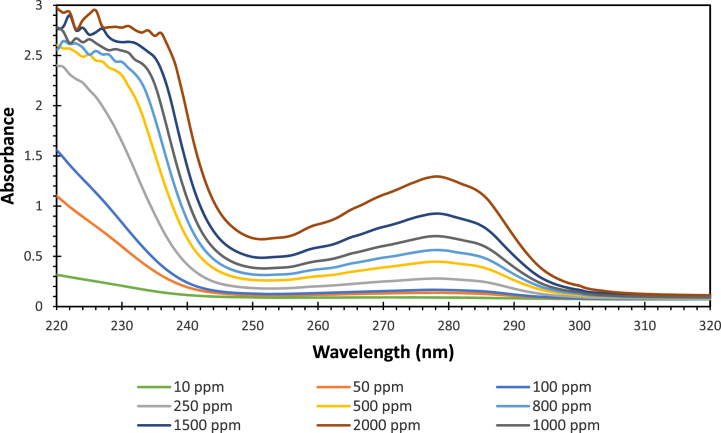
The UV–Vis spectrum of BSA- Water Standard samples.

A calibration curve for BSA in water was prepared to allow analysis of unknown samples containing different amounts of BSA in water. Nine different BSA in water standard samples were prepared and each was analyzed on the UV–vis. Fig. 1 shows the UV–vis spectrum of the BSA protein in water for the following concentrations: 10, 50, 100, 250, 500, 800, 1000, 1500, 2000 ppm.

**Fig. 2:**
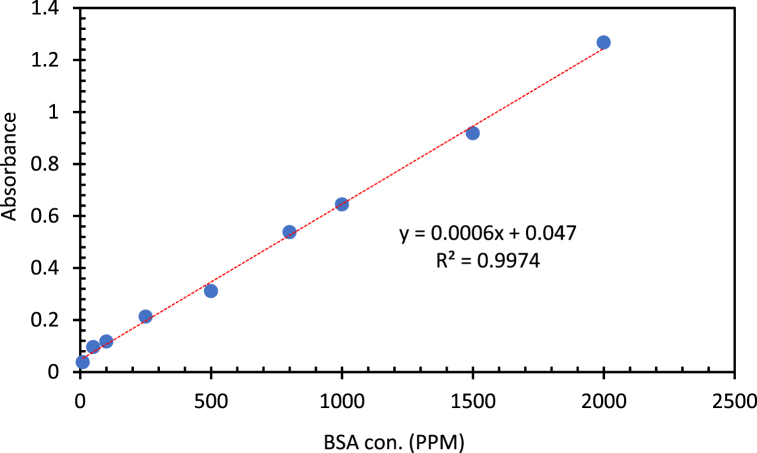
An example calibration curve obtained from sample optical density at 278 nm as a function of concentration of BSA in water.

It was observed in the literature that BSA interacts with light within a wavelength range of 260–280 nm, with a maximum at 278 nm [22]. Therefore, in order to obtain a calibration curve that can be used to identify the concentration of BSA in any unknown sample, the absorbances that correspond to the highest peak at 278 nm were recorded for the different concentration, and a linear calibration curve was plotted for absorbance versus BSA concentration in water. Fig. 2 shows an example calibration curve. The data points were fitted to a straight line and the straight-line equation is displayed showing an R^2^ = 0.9974.

### 3.2 Hansen dissolution tests

**Fig. 3:**
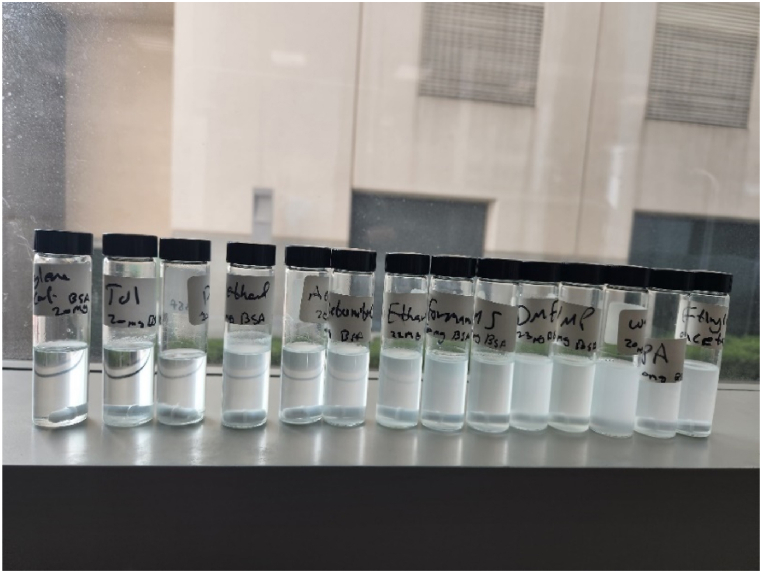
20 mg of BSA dissolved in 14 different solvents left for 24 h under continuous stirring.

The dissolution tests were carried out, where a measured amount (20–25 mg) of Bovine serum albumin (BSA) was dissolved in 10 ml of 14 different solvents (Fig. 3), with a minimum of three repeats for each solvent. Example raw data for one experimental run showing the initial, remaining and dissolved amount of BSA is shown in Table S1 in the supplementary information. The fourteen vials that contained the BSA were left overnight at room temperature under continuous stirring to ensure saturation was reached.

## 4 Results and discussion

### 4.1 Determination of BSA dissolved in different solvents using UV–vis spectrum

**Fig. 4:**
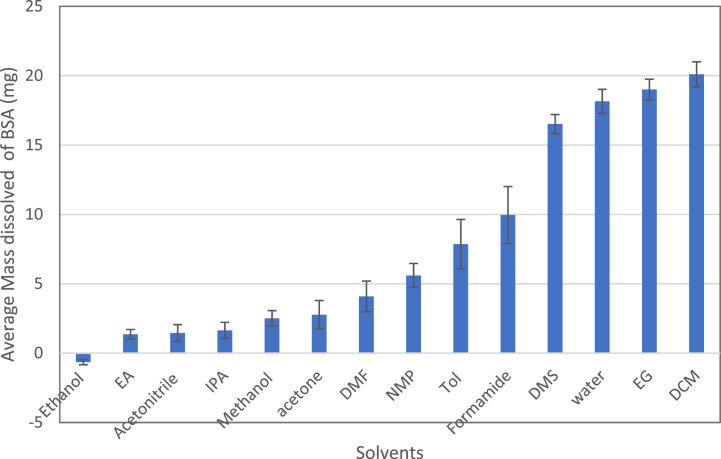
The average mass of BSA dissolved in mg in each organic solvent for three repeats (three experiments). The error-bars represent the standard error.

After 24 h of continuous stirring, the vials were centrifuged for 15 min under ambient temperature at a speed of 4000 RPM to allow pelleting of undissolved protein. The supernatant of all the 14 samples was decanted, and the remaining solid placed in a vacuum dryer at 25 °C for 24 h to allow complete evaporation of remaining solvent. The remaining solids were re-dissolved in 10 ml high purity water and analyzed using UV–vis. The spectrum at 278 nm was recorded and converted to concentrations of BSA in water using the standard curve (Fig. 2). The concentration of BSA in ppm (mg/L) was converted to a mass in mg, and since the initial mass of BSA dissolved in each vial was known and recorded, the amount that had dissolved in each vial (each solvent) could be calculated. The experiment was repeated three times and the average values of the amounts of BSA dissolved in each solvent in mg along with standard error are displayed in Fig. 4.

From Fig. 4, a criterion of ranking the different solvents was chosen based on the amount of BSA dissolved in each solvent. The four solvents DCM, water, DMS and EG showed an average dissolved amount of more than 10 mg, and therefore those four solvents were considered as very good solvents and ranked as 1. Formamide, Toluene and NMP all showed an average amount adsorbed above 5 mg, therefore they were considered good solvents but with lower ranking (ranked 2). DMF showed an amount adsorbed of around 4 mg and therefore it will be considered solvent but with lower ranking (ranked 3). All solvents showing ranking from 1 to 3 were considered as good solvents. However, those ranked 1 were the best performing solvents followed by those ranked 2 and finally 3. The rest of solvents will be considered as bad solvents (ranked zero).

### 4.2 Determination of Hansen solubility parameter (HSP)

**Fig. 5:**
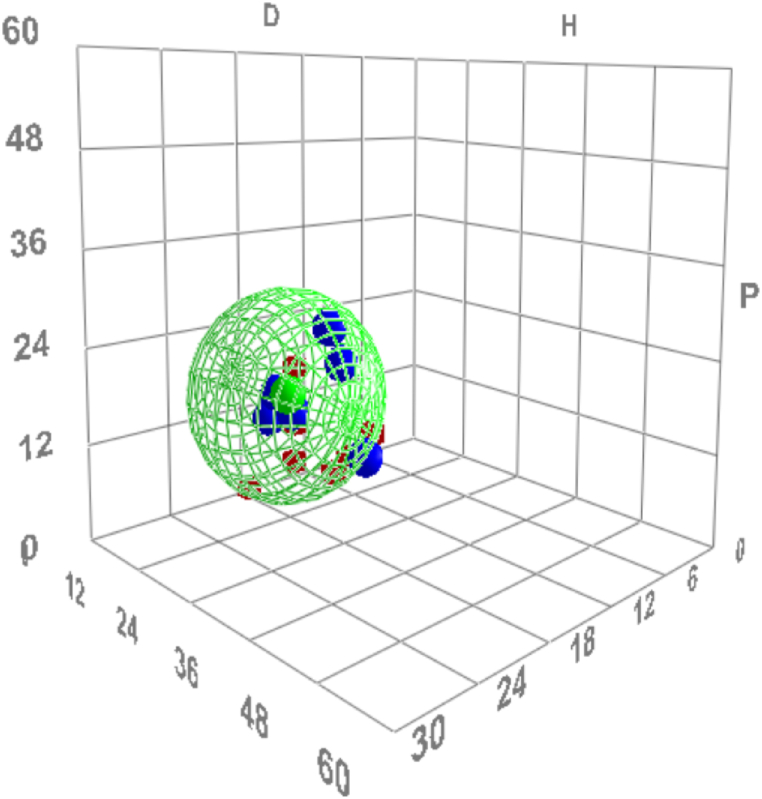
Hansen Solubility Sphere for BSA based on Run 3, which corresponds to the following HSP of BSA δd = 19.9, δp = 18.2, and δh = 17.5 MPa^0.5^.

HSP values were calculated using HSPiP software using a genetic algorithm. Based on the ranking of the solvents, the software was run several times to ensure a convergence to consistent values. Essentially, solvents of know solubility parameters are plotted in three-dimensional space, with axes of polar, non-polar and hydrogen bonding contributions. The genetic algorithm then goes through several iterations to fit a sphere around the solvents identified as good and excluding those identified as bad. The centre of the resultant sphere marks the coordinates for the solubility parameters of the unknown, in this case the BSA. The ranking of each solvent was input to the software and the results of the HSP are displayed in Table 1. The goodness of the fit in Run 1 was 0.786 and it showed three “wrong outs” DCM, EG, and toluene. Here, “wrong out” indicates that there was a disagreement between the ranking of the solvent as a good solvent from the experimental data and the software calculated RED value (i.e. above 1, indicating a non-solvent). To resolve the issue of “wrong out” results, normal practice is to change the ranking of one of the wrong out solvents to either consider it a non-solvent, or by eliminating it completely from the list of solvents. DCM and EG are definitely good solvents, and this was observed experimentally and they cannot be outside the solubility sphere. Therefore, to enhance the goodness of the fit, Toluene was ranked as zero (non-solvent) as also the standard deviation of this particular solvent was high, as shown in Fig. 4. The software was ran again and the goodness of the fit increased to 0.929, however it was still showing DCM as wrong out. A third run (run 3) to further enhance the goodness of the fit was done by eliminating DCM from the list. Run 3 showed a goodness of fit of 1 and hence HSP for the BSA will be considered for run 3. As a result, BSA has the following HSP based on this result: δd = 19.9, δp = 18.2, and δh = 17.5 MPa^0.5^ with a radius of interaction R_0_ of 12.6. Fig. 5 displays the Hansen solubility sphere of BSA based on Run 3. From run 2 to run 3, the HSP results did not change much, therefore eliminating the DCM solvent from the list of solvents did not significantly affect the HSP results. Table 2 lists the thirteen solvents used (DCM was eliminated as mentioned before), and their HSP values taken from Hansen data base, the ranking of each solvent as described previously and finally the RED value was calculated using the HSPiP software.

**Table 1:** The procedure of obtaining the HSP of BSA using HSPiP software, three different runs.

HSPiP Run Number	Ranking	δD	δP	δH	δT	R	Wrong in/out	Fit
1	DCM, DMS, water EG = 1 Toluene, Formamide, NMP = 2 DMF = 3 Rest = 0 In = 8 Out = 6 Total = 14	18.1	17.0	14.9	28.9	10.2	Wrong In = 0 Wrong Out = 3 DCM Toluene EG	0.786
2	DCM, DMS, water EG = 1 Formamide, NMP = 2 DMF = 3 Rest = 0 In = 7 Out = 7 Total = 14	20.0	19.7	17.0	32.8	14.0	Wrong In = 0 Wrong Out = 1 DCM	0.929
3	DMS, water EG = 1 Formamide, NMP = 2 DMF = 3 Rest = 0 DCM out of the list In = 6 Out = 7 Total = 13	19.9	18.2	17.5	32.1	12.6	Wrong In = 0 Wrong Out = 0	1

**Table 2:** List of solvents used in run 3 shown in Table 1, with the HSP of each solvent taken from the HSPiP software and ranking used with RED values obtained.

	Solvents	δD	δP	δH	Ranking	RED
1	Acetone	15.5	10.4	7.00	0	1.26
2	Acetonitrile	15.3	18.0	6.10	0	1.17
3	Dimethyl Formamide	17.4	13.7	11.3	3	0.75
4	Dimethyl Sulfoxide	18.4	16.4	10.2	1	0.66
5	Ethanol 99.9 %	15.8	8.80	19.4	0	1.02
6	Ethyl Acetate	15.8	5.30	7.20	0	1.46
7	Methanol	14.7	12.3	22.3	0	1.04
8	n-Methyl Pyrrolidone	18.0	12.3	7.2	2	0.99
9	Toluene	18.0	1.4	2.0	0	1.81
10	Ethylene Glycol	17.0	11.0	26.0	1	0.99
11	2-Propanol	15.8	6.10	16.4	0	1.17
12	Water 1 % Sol In	15.1	20.4	16.5	1	0.82
13	Formamide	17.2	26.2	19.0	2	0.78

### 4.3 Comparing the HSPs of BSA obtained in this work with other literature values

Fang et al. [3] estimated the HSP of surface adsorbed protein using static adsorption data. The HSP was obtained via optimizing the HSP distance values for a set of polymers with BSA based on a proposed straight-line relation between the natural logarithm of the adsorbed amounts and HSP distance. The HSP estimated by this method for surface adsorbed BSA was as follows: δ_d_ = 22.94, δ_p_ = 11.06, and δ_h_ = 20.76 MPa^0.5^. These values give a total HSP δ_tot_ = 32.85 MPa^0.5^. These values are not in very good agreement with the results obtained in this work, and this likely to be because the HSP values from Fang et al.’s work were for a surface adsorbed protein, while the HSP calculated here are for native BSA in solution. Fang et al. suggested in their work that surface adsorbed proteins are partially denatured and expose their hydrophobic core toward the polymer surface. This would result in very different solubility parameters to the same proteins when in solution.

**Fig. 6:**
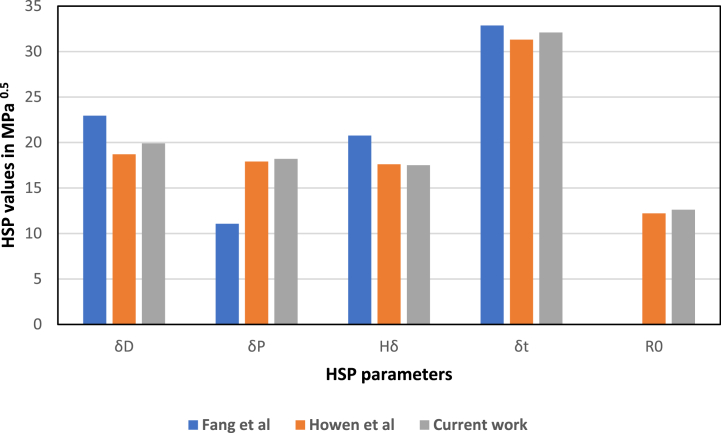
Comparison between the HSPs of native BSA protein from the current work, the work of Fang et al., 2019 [3] for surface adsorbed BSA and the work of Houen et al., 1996 [19] for native BSA protein.

Houen et al. [19] reported the BSA solubility in 19 different solvents calculated using amino acid analysis. The organic solvents used in their work are listed in Table 2, with their obtained solubility in mg/L. In this work, HSP parameters were calculated from the solubility data of Houen et al. and according to their commentary of their experimental observations (Table 3), the solvents were ranked accordingly, and the ranking was fed to the software to estimate the HSP of BSA protein. The HSP results obtained for this case was as follows: δ_d_ = 18.7, δ_p_ = 17.9, and δ_h_ = 17.6 MPa^0.5^, with δ_tot_ = 31.3 MPa^0.5^ and a center of sphere of radius R_0_ = 12.2. Those values are in better agreement with the values obtained via the proposed UV–vis ranking method in this work. Fig. 6 compares the HSP values obtained in this work with the HSP values of surface adsorbed protein and native protein estimated by Fang et al. and Houen et al. respectively. The HSP from the work of Houen et al. are in better agreement with the HSP found in this work compared to the work of Fang et al.

**Table 3:** Solubility of BSA in various organic solvents by Houen et al., 1996 [19].

No.	solvents	Solubility (mg/L)	Comments
1	Acetic acid	0.02	Clear supernatant
2	Acetonitrile	0.02	Clear supernatant
3	Benzene	0.04	Clear supernatant
4	1-Butanol	0.03	Clear supernatant
5	1-Chlorobutane	0.01	Clear supernatant
6	Chloroform	0.12	Cloudy supernatant
7	Dimethyl Sulfoxide	5.12	Clear supernatant
8	N- Dimethylformamide	0.01	Clear supernatant
9	1,4-Dioxane	0.01	Clear supernatant
10	Ethanol	0.01	Clear supernatant
11	Formic Acid	>50	All dissolved
12	Glycerol	20	Clear supernatant, half dissolved
13	Heptane	0.00	Clear supernatant
14	Isoamyl Alcohol	0.02	Clear supernatant
15	3-Mercaptoproponice acid	5.45	Clear supernatant, some dissolved
16	Methanol	0.01	Clear supernatant
17	1-Propanol	0.03	Clear supernatant
18	Triethanolamine	0.70	Clear supernatant
19	Trifluoroacetic acid	>50	All dissolved

## 5 Conclusions

A new simple procedure to properly rank the solubility of BSA protein in a number of solvents as an alternative to visual observation in dissolution tests proposed by Hansen is presented. This method is based on analyzing samples using UV–vis spectrophotometer with the use of one calibration curve for standard samples of BSA in water only. This method can be applied to any other material that is known to have a high solubility in water, including other proteins and macromolecules, provided they have a clear peak in the UV/Vis range. This method will provide a qualitative result only that will help ranking the solvents as good and bad ones so that the ranking can be used in HSPiP software for HSP estimation. The HSP of the BSA obtained in this work is comparable to literature values that were obtained using different methods, especially to the none adsorbed BSA protein.

## CRediT authorship contribution statement

**Neveen AlQasas:** Conceptualization, Data curation, Formal analysis, Investigation, Methodology, Writing – original draft, Writing – review & editing. **Daniel Johnson:** Conceptualization, Formal analysis, Funding acquisition, Methodology, Project administration, Resources, Supervision, Writing – review & editing.

## Declaration of competing interest

The authors declare that they have no known competing financial interests or personal relationships that could have appeared to influence the work reported in this paper.
