# Supplementary material for: Determination of Hansen solubility parameters of water-soluble proteins using UV–vis spectrophotometry
Source: Heliyon. 2023 Oct 29;9(11):e21403. doi: 10.1016/j.heliyon.2023.e21403 (PMC10643263; doi:10.1016/j.heliyon.2023.e21403)
Supplement: Multimedia component 1 [file mmc1.docx]

| **Solvents** | **Absorb. at 278nm** | **Conc. Of BSA in ppm (mg/L)** | **m_r_ BSA (mg)** | **m_o_ BSA (mg)** | **m_d_ BSA (mg)** |
| --- | --- | --- | --- | --- | --- |
| DMF | 0.9776 | 1551.6628 | 15.516628 | 22 | 6.483372 |
| NMP | 0.8187 | 1287.01485 | 12.8701485 | 22 | 9.1298515 |
| water | 0.1473 | 168.79815 | 1.6879815 | 21 | 19.3120185 |
| EG | 0.1381 | 153.47555 | 1.5347555 | 22 | 20.4652445 |
| DMS | 0.3647 | 530.87785 | 5.3087785 | 20 | 14.6912215 |
| Ethanol | 1.4499 | 2338.27845 | 23.3827845 | 22 | -1.3827845 |
| DCM | 0.23 | 306.535 | 3.06535 | 25 | 21.93465 |
| Methanol | 1.011 | 1607.2905 | 16.072905 | 21 | 4.927095 |
| Acetonitrile | 1.007 | 1600.6285 | 16.006285 | 20 | 3.993715 |
| Formamide | 0.3447 | 497.56785 | 4.9756785 | 21 | 16.0243215 |
| IPA | 1.3445 | 2162.73475 | 21.6273475 | 22 | 0.3726525 |
| EA | 1.2729 | 2043.48495 | 20.4348495 | 21 | 0.5651505 |
| Toluene | 0.4944 | 746.8932 | 7.468932 | 22 | 14.531068 |
| Acetone | 0.9139 | 1445.57045 | 14.4557045 | 21 | 6.5442955 |

Supplementary Table S1: A sample of raw data of one of the experiments, showing the initial, remaining and dissolved amount of BSA for each solvent.

Where m_r_ , m_0_, and m_d_ stands for the remaining, the initial and the dissolved amounts of BSA in mg. m_0_ here shows the initial amounts of BSA added to each vial that contains a different solvent as shown in the above table.
